# Supplementary material for: SQANTI-SIM: a simulator of controlled transcript novelty for lrRNA-seq benchmark
Source: bioRxiv. 2023 Aug 24:2023.08.23.554392. Preprint. [Version 1] doi: 10.1101/2023.08.23.554392 (PMC10473693; doi:10.1101/2023.08.23.554392)
Supplement: Supplement 2 [file NIHPP2023.08.23.554392v1-supplement-2.pdf]

bioRxiv preprint doi: https://doi.org/10.1101/2023.08.23.554392; this version posted August 24, 2023. The copyright holder for this preprint (which was not certified by peer review) is the author/funder, who has granted bioRxiv a license to display the preprint in perpetuity. It is made available under aCC-BY 4.0 International license.

Appendix A Supplementary Figures

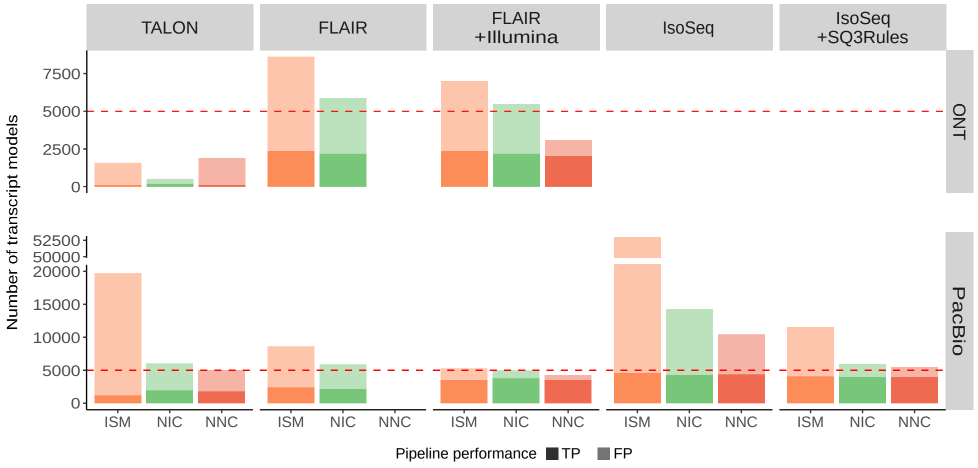

Fig. A1 Number of detected true (TP) and false positives (FP) for different types of novelty (ISM, NIC, and NNC).

bioRxiv preprint doi: https://doi.org/10.1101/2023.08.23.554392; this version posted August 24, 2023. The copyright holder for this preprint (which was not certified by peer review) is the author/funder, who has granted bioRxiv a license to display the preprint in perpetuity. It is made available under aCC-BY 4.0 International license.

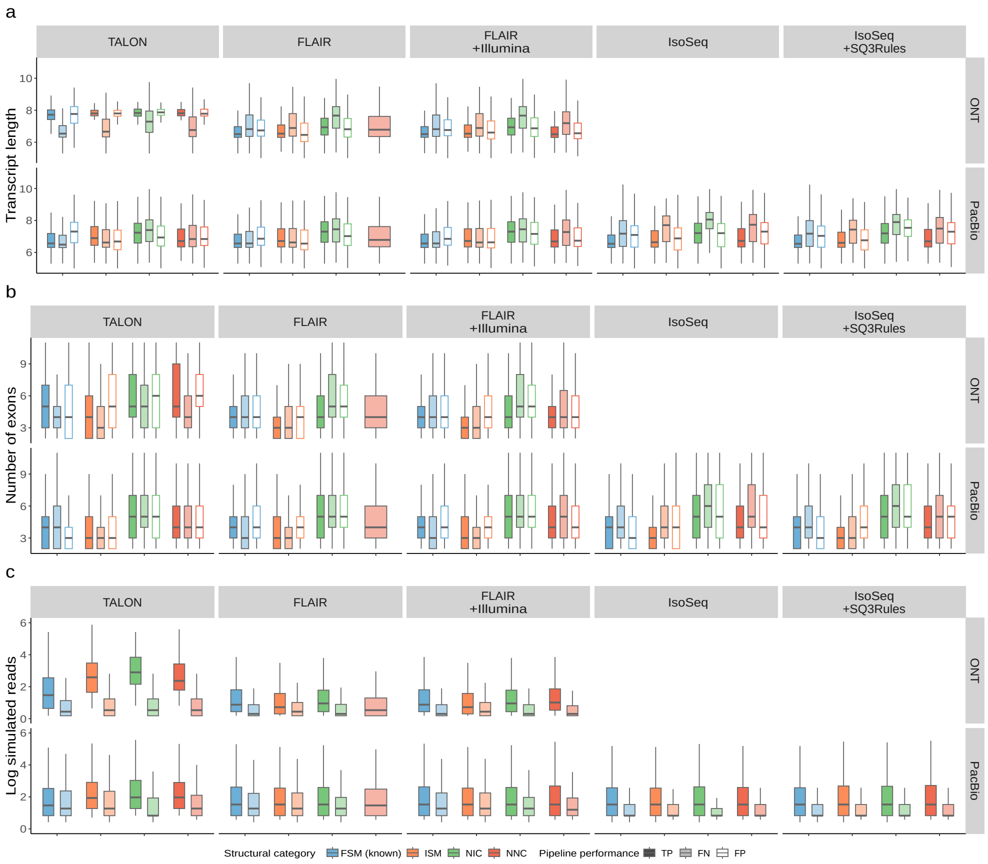

Fig. A2 Relationship between true positives (TP), false negatives (FN), and false positives (FP) with (**a**) transcript length, (**b**) number of exons, and (**c**) simulated expression level.

bioRxiv preprint doi: https://doi.org/10.1101/2023.08.23.554392; this version posted August 24, 2023. The copyright holder for this preprint (which was not certified by peer review) is the author/funder, who has granted bioRxiv a license to display the preprint in perpetuity. It is made available under aCC-BY 4.0 International license.

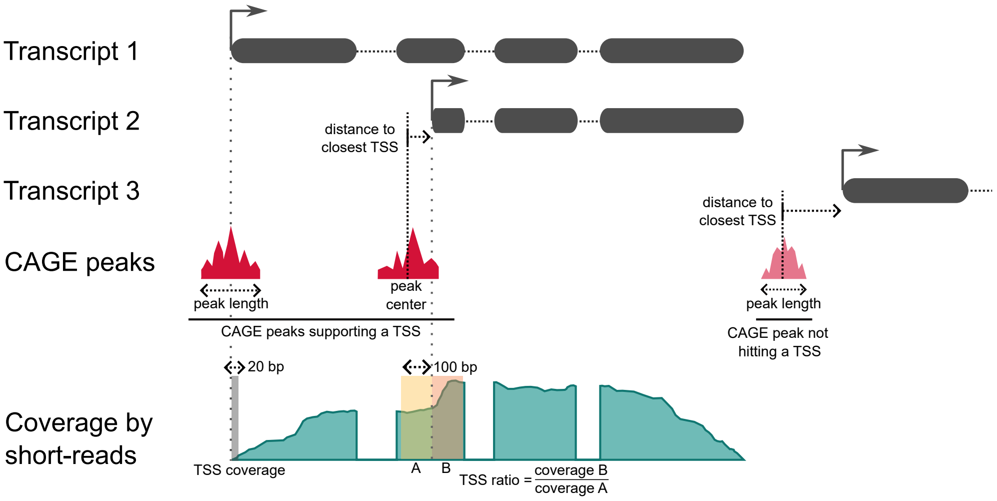

Fig. A3 SQANTI-SIM characterization of CAGE peak data.
